# Recyclable and Flexible Starch-Ag Networks and Its Application in Joint Sensor

**DOI:** 10.1186/s11671-019-2957-3

**Published:** 2019-04-05

**Authors:** Sai Liu, Cong Chen, Dongwei Zhang, Guanping Dong, Dongfeng Zheng, Yue Jiang, Guofu Zhou, Jun-Ming Liu, Krzysztof Kempa, Jinwei Gao

**Affiliations:** 1 Institute for Advanced Materials, South China Academy of Advanced Optoelectronics, South China Normal University, Guangzhou, 510006 China; 2 School of Mechanical and Automotive Engineering, South China University of Technology, Guangzhou, 510640 China; 3 Guangdong Provincial Key Laboratory of Optical Information Materials and Technology and Institute of Electronic Paper Displays, South China Academy of Advanced Optoelectronics, South China Normal University, Guangzhou, 510006 China; 4 Laboratory of Solid State Microstructures, Nanjing University, Nanjing, 210093 China; 5 Department of Physics, Boston College, Chestnut Hill, MA 02467 USA

**Keywords:** Starch-ANs, Recyclability, Optoelectronics, Ultra-smooth morphology, Sensor

## Abstract

Flexible transparent conductive electrodes are essential component for flexible optoelectronic devices and have been extensively studied in recent years, while most of the researches are focusing on the electrode itself, few topics in material green and recyclability. In this paper, we demonstrate a high-performance transparent conductive electrode (TCE), based on our previous cracking technology, combined with a green and recyclable substrate, a starch film. It not only shows low *R*_s_ (less than 1.0 Ω sq^−1^), high transparency (> 82%, figure of merit ≈ 10,000), but also provides an ultra-smooth morphology and recyclability. Furthermore, a series of biosensors on human joints are demonstrated, showing great sensitivity and mechanical stability.

**Electronic supplementary material:**

The online version of this article (10.1186/s11671-019-2957-3) contains supplementary material, which is available to authorized users.

## Introduction

Currently, electronic devices have been experiencing many new challenges, such as compatibility, mechanical flexibility, and eco-friendly manner [1–5]. Among those, transparent conductive electrode (TCE) as an important component of those devices is also facing new challenges, like high optical transmittance, low resistance, flexibility, biocompatibility [6], low-cost [7], and the recyclability [8]. Currently, indium tin oxide (ITO) [9] is the widely used TCE, which is a continuous and chemically stable film. However, its fragility induced by the metal oxide and the large expense because of rare metal highly limit its future development. On the other hand, graphene/metal grid [10, 11], for example, metal networks [12, 13] and metal nanowires [14–19], is facing serious adhesiveness and roughness problems. In addition, their high synthesizing cost and the impossibility to recycle make them detained in the laboratory.

In comparison, a series of TCEs based on crack-nanonetwork (CNN) [20] have been invented by our group, which presenting brilliant optoelectronic properties, high figure of merit, and the flexibility. With electroplating technology [21], we further realized the fully wet fabricated CNN based on UV glue with ultra-low sheet resistance (0.13 Ω sq^−1^) and smooth morphology [22]. Currently, all of the substrates are based on the intrinsic non-degradation polymers, restraining the recycling of precious metal, like Ag and Au. Starch film is a transparent and flexible substrate material, and more importantly, it is an eco-friendly material and could be degraded in water. Jeong et al. [23] added PVA into a starch film and fabricated a flexible and disposable TCE; thus, it shows great potential of starch film as substrates.

Herein, we took the advantage of water degradability of starch film [24, 25] and fabricated a recyclable TCE, starch-Ag networks (SANs), through embedding our previously reported crack Ag networks in starch film. Via electroplating, we decreased the sheet resistance (*R*_s_) to less than 1.0 Ω sq^−1^ along with highly optical transparency (> 82%) and high figure of merit (*F*) of over 10,000. Moreover, due to the peeling off fabrication process and self-supporting network [26], SAN presents good flexibility, low surface roughness, and the recyclability. Besides, SAN was used to demonstrate its application in biosensors in human joint with good sensitivity and mechanical stability.

## Methods

### Fabrication Process

**Fig. 1:**
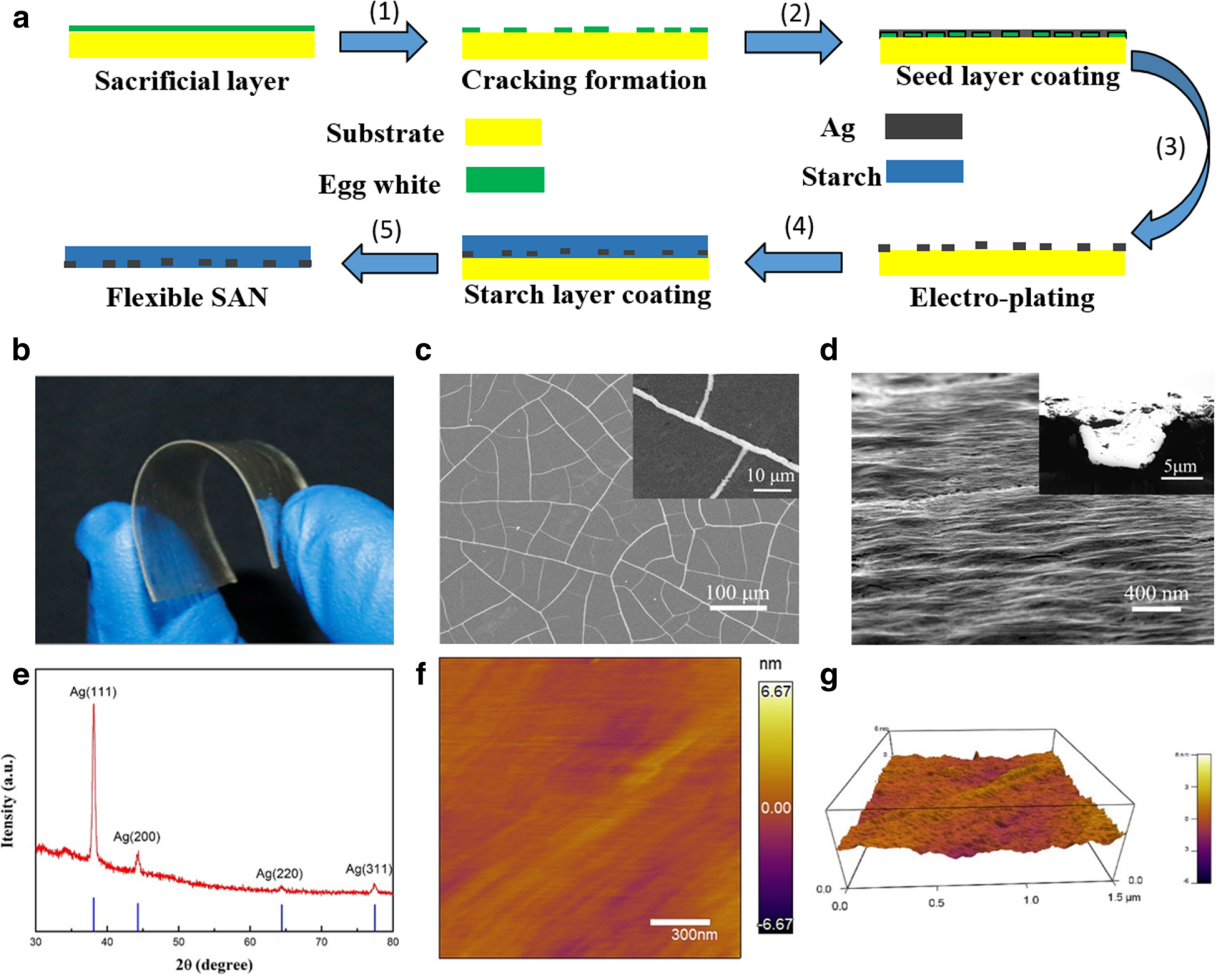
Fabrications and characterizations of SAN samples. **a** Fabrication processes. **b** Flexibility demonstration of a SAN sample. **c** SEM images. Inset shows an enlarged metallic network. **d** Tilted (60°) SEM image of the embedded Ag networks. Inset is the cross-sectional view of the Ag network. **e** XRD spectra. **f**, **g** AFM images of the surface morphology

Figure 1a schematically presents the fabrication process of the SANs. Step 1 is to prepare the network template with the method invented by our group [27]. Firstly, the egg white self-cracks during drying process thereby form the channel networks. After the deposition of Ag seed layer with sputtering (step 2), the sacrificial layer is washed away. Subsequently, a dense layer of Ag is further deposited on the surface of seed layer metal network via electroplating deposition (step 3). In step 4, the Ag networks are covered with starch film by dip coating the prepared starch solution and drying naturally. Finally, the Ag networks embedded in starch are peeled off from quartz. On account that the gelatinization temperature of normal starch is intrinsically high (usually more than 90 °C) [28], herein starch mechanical property is enhanced by its room temperature gelatinization.

### Preparation of the Sacrificial Template

Self-cracking materials are a mixture of egg white and deionized water (3:1 by volume). A cracking template is obtained by dip coating above solution on a glass (50 mm × 50 mm), then drying in air about 10 mins, and finally, the self-cracking process occurs.

### Ag Seed Layer Deposition

Sputtering (AJA International ATC Orion 8, USA) was used to deposit Ag seed layers (≈ 60 nm) on self-cracking template. Then, the sacrificial layer is removed by rinsing in deionized water.

### Electroplating of Ag Networks Based on CNN Layers

One hundred-milliliter Ag electroplate liquid composed of 4 g AgNO_3_, 22.5 g Na_2_S_2_O_3_·5H_2_O, and 4 g KHSO_3_ in deionized water was used for the electroplating deposition. A homemade plating bath is used in the process, with a seed layer as the cathode and a Ag bar (40 mm × 40 mm) as the anode. The current for the electroplating deposition is 10 mA. We changed the thickness of the film by controlling the plating time. Finally, the Ag networks were rinsed in deionized water.

### Fabrication of a Starch TCE

The starch solution, composed of 12.5 g corn starch, 1.25 g glycerinum (10 wt%) in 100 ml deionized water, was prepared at 60 °C on a hot plate, with stirring at 500 rpm for 30 min. The bubbles were removed from the starch solution in vacuum environment for 2 h. Four-milliliter starch solution was dip coated on the electroplating TCE and then dried in air for about 20 h under 30–40% RH and 25 °C.

### Transfer of Ag Networks

The starch-Ag network film was immersed in DI water under 25 °C for 2 h. Then, the starch layer is dissolved, and finally, the freestanding Ag network was obtained.

### Characterizations

The morphologies of samples were conducted by a SEM (ZEISS Gemini 500, Garl Zeiss, Germany), photographic camera, and atomic force microscope (AFM) (Cypher, Asylum Research). The crystallinity and phase information of the metal particles were determined by an X-ray diffraction system (PAN analytical X’Pert-Pro MPD PW 3040/60 XRD with Cu-Kα1 radiation, Netherlands). Optical transmittance was measured using an integrating sphere system (Ocean Optics, USA). Sheet resistance of samples was measured by a van der Pauw method, with four silver paste contacts deposited at the corners of a square sample (20 mm × 20 mm), recording with a Keithley 2400 SourceMeter (Keithley, USA). Two-probe resistance method is conducted in a bending test (Additional file 1).

## Results and Discussion

### Sample Morphologies

Figure 1b is a schematic figure of the obtained SAN sample, showing a good flexibility and transparency. The SEM image of the metallic network is shown in Fig. 1c, with an average width and height of the Ag networks 2.5 μm and 1 μm respectively, and the inter-thread spacing in the range of 30 to 60 μm. The inset in Fig. 1c clearly displays the detailed morphology of the metal networks. The surface morphology of the SAN film is shown in Fig. 1d, with the inset of cross-sectional image, proving that the Ag networks have been successfully embedded into the starch film and exhibiting a smooth morphology. In addition, the height of Ag networks could be easily modulated by changing the concentration of the electroplating liquid, anode area, and distance between an anode and cathode in the electroplating deposition process [29], while the width of the networks and the inter-space can be controlled by varying the sacrificial material, concentration, and cracking temperature, as reported in our previous work [30]. The crystallinity of SAN was characterized by X-ray diffraction (XRD) (Fig. 1e), which exhibits the (200), (220), and (311) planes of Ag, and no impurity detected. Atomic force microscopy (AFM) images in Fig. 1f, g confirmed an ultra-smooth surface with an extremely low root-mean-square (RMS) roughness of ~ 0.521 nm.

### Optical and Mechanical Performance

**Fig. 2:**
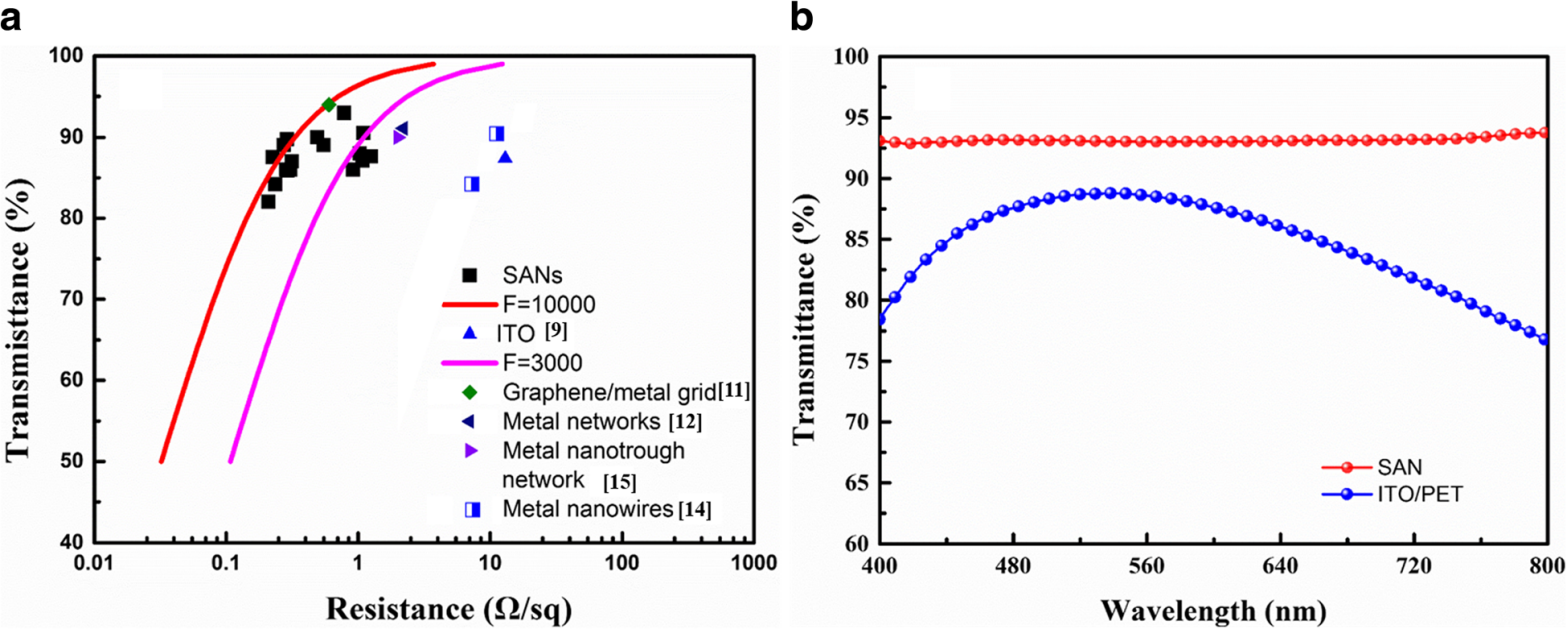
Optoelectronic properties of the metallic networks. **a** Optical transmittance of metallic networks as a function of sheet resistance. **b** Transmittance versus wavelength of the SAN and a ITO/PET sample

Figure 2a shows the transmittance (*T*) versus the sheet resistance (*R*_s_) plots, comparing the optoelectronic properties of the SAN with other reported TCEs [5, 6, 31–36] and a commercial ITO film (150 nm thick, Liaoning Huite Photoelectric Technology). A figure of merit (*F*), shown as lines, is determined by fitting the equation in [37]. Our SAN shows very good optoelectronic properties with the high transparency (82–93%) and low sheet resistance (0.2–1.0 Ωsq^−1^, with *F* ranging from 3000 to 10,000) based on different cracking templates [38]. These data are significantly better than those of conventional ITO and other grid TCEs, which could be ascribed to the excellent crystallinity of Ag, the continuous morphology, and the appropriate network structure. Figure 2b shows optical transmittance of the SAN and ITO/PET (150 nm thick, Liaoning Huite Photoelectric Technology Co., Ltd.). It is clear that the optical transmittance of the SAN (~ 93%) is much higher than that of ITO/PET (77~88%) in the entire visible spectrum.

### Recyclable

**Fig. 3:**
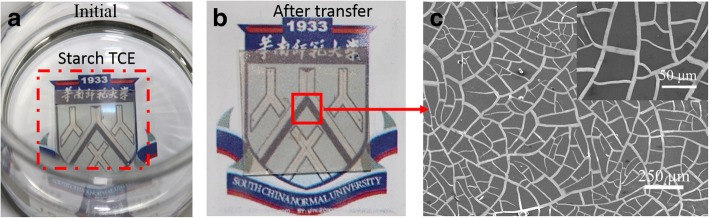
Recyclability test of a SAN in water: **a** original and **b** after transfer. **c** SEM images of recycled Ag networks

Starch is not only a green material and non-toxic for human beings or the environment, but also a biodegradable material, as well as easily removed by water [39]. These properties, therefore, endow the SAN a recyclable material as illustrated in Fig. 3. A piece of used SAN film was immersed into the water (Fig. 3a), and 2 h later, most of the starch substrate was degraded, and water turned into opaque state. The obtained freestanding Ag networks was washed with water to remove residue starch and then transferred onto a piece of a ITO glass and dried in a drying box (Fig. 3b). Figure 3c shows SEM images of the recycled Ag networks. It is worth mentioning that the recycling process keeps the integrity of the Ag networks due to its self-supporting property, rendering the recyclability of the process and finally reducing the overall cost and the environmental impact, comparing with the TCEs based on the non-degradable and non-recyclable plastic substrates [5, 9, 40–42].

### Sensing Performance of the SAN

**Fig. 4:**
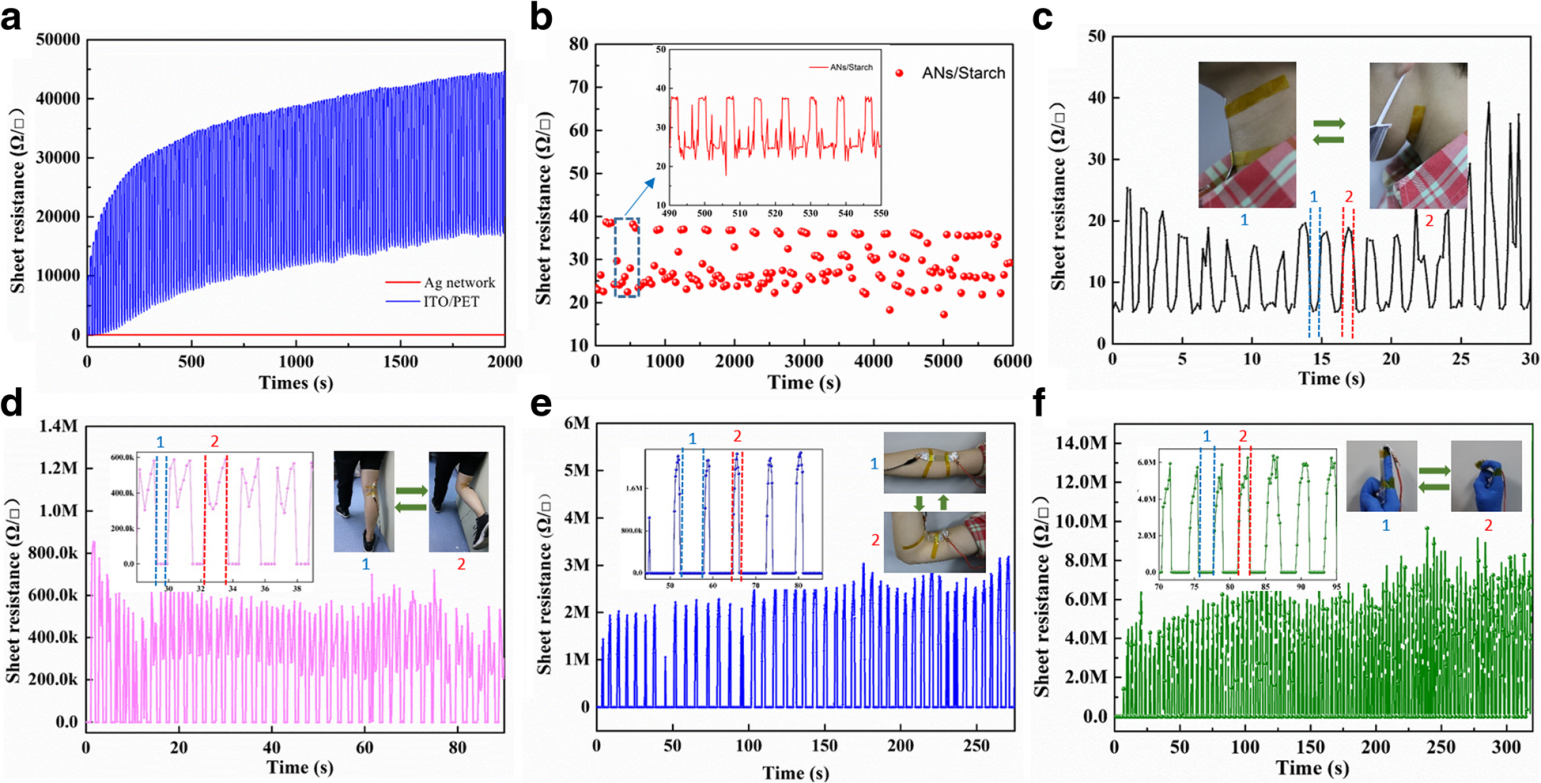
Flexibility demonstration of the SAN based sensor. **a** Comparison of sheet resistance as a function of bending time. **b** An enlarged figure of **a**; inset shows the detailed variation of sheet resistance of the SAN sensor from 490 to 550 s. **c**–**f** Characterization of sensors bending at different part of human body: **c** neck, **d** knee, **e** elbow, and **f** finger. Insets: photographs of the sensors attached to different part of human body

The flexibility of the SAN was characterized under bending in comparison with a ITO/PET sample. The *R*_s_ of ITO/PET raised significantly (~ 35,000 Ω sq^−1^) within a thousand bending cycles (Fig. 4a), whereas the *R*_s_ of the SAN fluctuates around 30 Ω sq^−1^, showing an excellent mechanical stability (Fig. 4a, b). Simultaneously, a periodic fluctuation of *R*_s_ was observed when the SAN was bent (from 24 to 38 Ω sq^−1^) as shown in the inset of Fig. 4b, which is suggestive of its potential application on mechanical sensor [43–47]. Accordingly, a series of simple joint sensors were designed and fabricated [48–51]. The SAN with two narrow silver paste lines along edges to give better contact was sandwiched between two pieces of PET films, which was attached on the joint the of neck, knee, elbow, finger, respectively. The motion-dependent response of these sensors was recorded by a two-probe resistance measurement setup. When the joints were in bending stage, the *R*_s_ of the sensor changed correspondingly as demonstrated in Fig. 4c–f. When the SAN was under tensile stress at different parts of the body, the output signal varied in a wide range: on the neck, *R*_s_ is about 20–30 Ω sq^−1^ (Fig. 4c), on knee 400–800 KΩ sq^−1^(Fig. 4d), on elbow 2–3 MΩ sq^−1^ (Fig. 4e), and on finger 4–8 MΩ sq^−1^ (Fig. 4f). These differences are possibly associated with the magnitude of movement and indicate that the performance of joint SAN sensor are location-dependent [52].

**Fig. 5:**
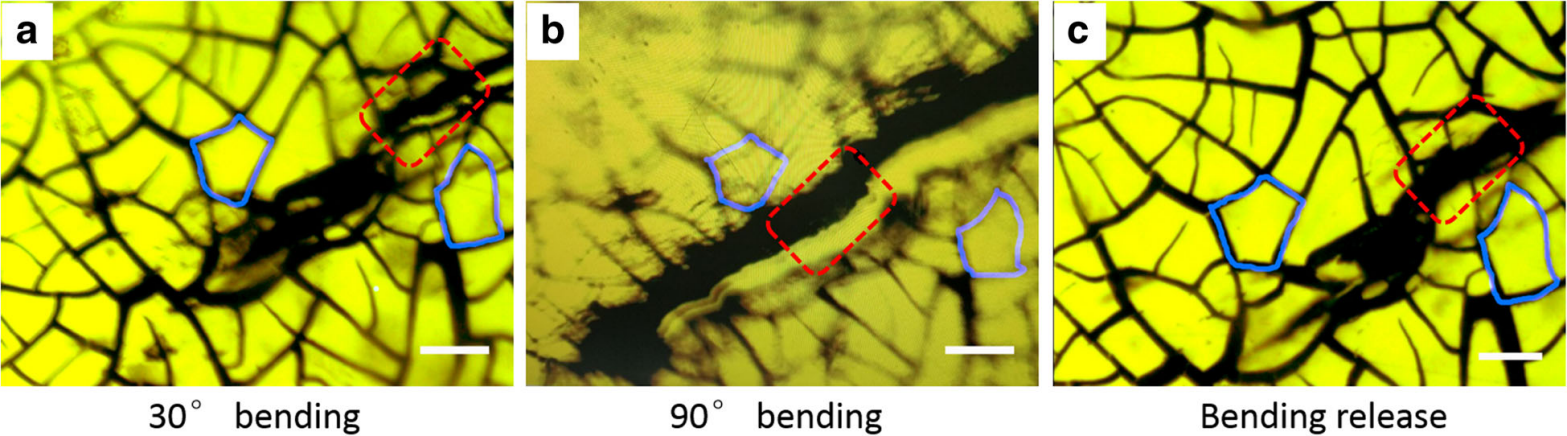
Working mechanism of the SAN sensor at different stages: **a** 30° bending, **b** 90° bending, and **c** bending release. Scale bars in figures are 50 μm

Figure 5 demonstrates the working mechanism of the SAN sensors, with blue lines locating the identical area. When the bending is limited to 30^o^, a subtle cracking was observed as indicated by the red rectangle in Fig. 5a. In spite of the difficulty to obtain a well-focused image, when the bending angle increased to 90^o^, the distance of this cracking slit was found further widened as well as its elongation (Fig. 5b). The re-flattening process, however, induced the recovery of the cracking which could barely be seen (Fig. 5c). In the meantime, the resistance of the SAN almost fully recovered to its initial state, as shown in Fig. 4a–d. Hence, the periodic variation of resistance during bending is attributed to the dynamic change of Ag network connection.

## Conclusion

In conclusion, we have developed high-performance recyclable metallic networks, by combining the cracking network with starch substrates. The corresponding figure of merit of the resulting metallic network exceeds 10,000 with the sheet resistance (*R*_s_) to less than 1.0 Ω sq^−1^ along with highly optical transparency (> 82%). Most importantly, the metallic network presents good flexibility, low surface roughness, and recyclability. Finally, a series of biosensors have been demonstrated showing good performance.

## Additional file

Additional file 1: **Figure S1.** Images of SAN with different glycerin mass ratio. a) wt5%, b) wt10%, c) wt20%. **Figure S2.** (a) Photograph of the setup for bending test, which includes mobile station (1), controller of the mobile station (2, 3), voltage transformer (4), (5) Keithley 2400. (b) Schematic diagram of the bending test. **Table S1.** Comparison of sheet resistance as a recovering of time: original and after transfer. (DOCX 1585 kb)

## Abbreviations

AFM: Atomic force microscopy
CNN: Crack-nanonetwork
*F*: Figure of merit
ITO: Indium tin oxide
RMS: Root-mean-square
*R*_s_: Sheet resistance
SAN: Starch-Ag network
SEM: Scanning electron microscope
*T*: Transmittance
TCE: Transparent conductive electrode
XRD: X-ray diffraction
