# Supplementary material for: Recyclable and Flexible Starch-Ag Networks and Its Application in Joint Sensor
Source: Nanoscale Res Lett. 2019 Apr 5;14:127. doi: 10.1186/s11671-019-2957-3 (PMC6450995; doi:10.1186/s11671-019-2957-3)
Supplement: Supplementary file 1 — Figure S1. Images of SAN with different glycerin mass ratio. a) wt5%, b) wt10%, c) wt20%. Figure S2. (a) Photograph of the setup for bending test, which includes mobile station (1), controller of the mobile station (2, 3), voltage transformer (4), (5) Keithley 2400. (b) Schematic diagram of the bending test. Table S1. Comparison of sheet resistance as a recovering of time: original and after transfer. (DOCX 1585 kb) [file 11671_2019_2957_MOESM1_ESM.docx]

Electronic Supplementary Information

**Recyclable and Flexible Starch-Ag Networks and Its Application in Joint Sensor**

Sai Liu, ^†^ Cong Chen, ^†^ Dongwei Zhang, Guanping Dong, Dongfeng Zheng, Yue Jiang, ^*^ Guofu Zhou, Jun-Ming Liu, Krzysztof Kempa, Jinwei Gao^*^

1. **Figure S1.** Images of SAN with different glycerin mass ratio. a) wt5%, b) wt10%, c) wt20%.
2. **Figure S2.** (a) Photograph of the set-up for bending test, which includes mobile station (1), controller of the mobile station (2, 3), voltage transformer (4), (5) Keithley 2400. (b) Schematic diagram of the bending test.
3. **Table S1.** Comparison of sheet resistance as a recovering of time: original and after transfer.


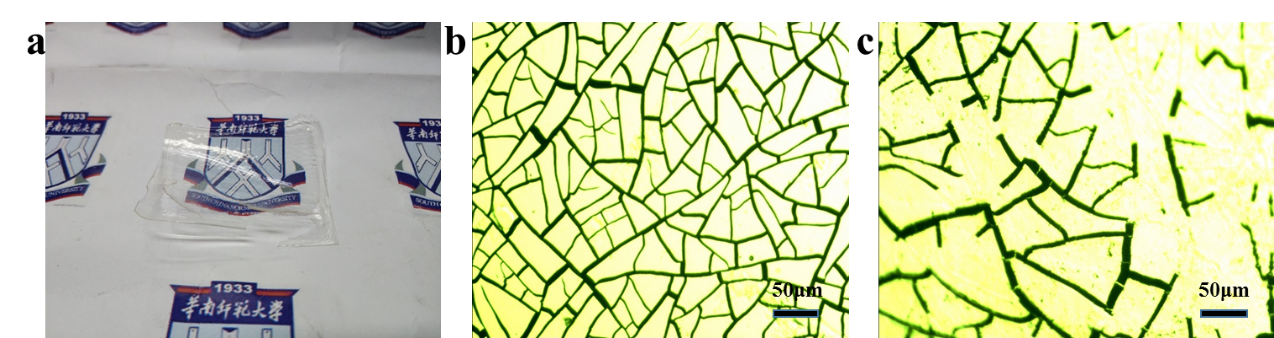


**Figure S1.** Images of SAN with different glycerin mass ratio. a) wt5%, b) wt10%, c) wt20%.


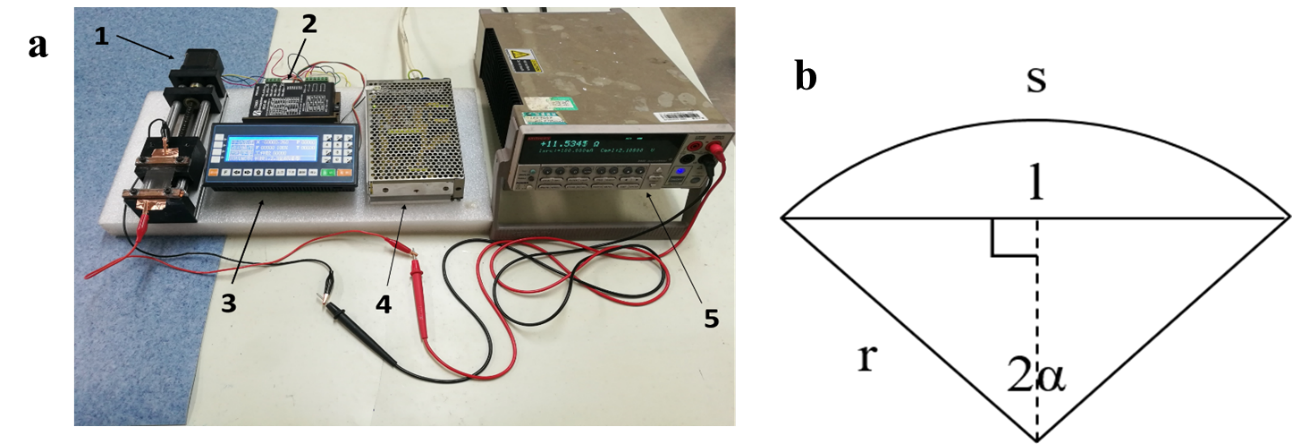


**Figure S2.** (a) Photograph of the set-up for bending test, which includes mobile station (1), controller of the mobile station (2, 3), voltage transformer (4), (5) Keithley 2400. (b) Schematic diagram of the bending test.

**Table S1.** Comparison of sheet resistance as a recovering of time: original and after transfer.

| Number | Original (Ω/sq) | After 2h soaking (Ω/sq) |
| --- | --- | --- |
| 1 | 0.45 | 0.54 |
| 2 | 0.4725 | 0.72 |
| 3 | 0.9225 | 1.26 |
| 4 | 1.1925 | 1.62 |
